# High fat feeding affects the number of GPR120 cells and enteroendocrine cells in the mouse stomach

**DOI:** 10.3389/fphys.2015.00053

**Published:** 2015-02-27

**Authors:** Patricia Widmayer, Hannah Goldschmid, Helena Henkel, Markus Küper, Alfred Königsrainer, Heinz Breer

**Affiliations:** ^1^Institute of Physiology, University of HohenheimStuttgart, Germany; ^2^Visceral and Transplant Surgery, University Hospital for GeneralTübingen, Germany

**Keywords:** fatty acid receptor, FFAR, diet induced obesity, stomach, lipid sensing cells

## Abstract

Long-term intake of dietary fat is supposed to be associated with adaptive reactions of the organism and it is assumptive that this is particularly true for fat responsive epithelial cells in the mucosa of the gastrointestinal tract. Recent studies suggest that epithelial cells expressing the receptor for medium and long chain fatty acids, GPR120 (FFAR4), may operate as fat sensors. Changes in expression level and/or cell density are supposed to be accompanied with a consumption of high fat (HF) diet. To assess whether feeding a HF diet might impact on the expression of fatty acid receptors or the number of lipid sensing cells as well as enteroendocrine cell populations, gastric tissue samples of non-obese and obese mice were compared using a real time PCR and immunohistochemical approach. In this study, we have identified GPR120 cells in the corpus region of the mouse stomach which appeared to be brush cells. Monitoring the effect of HF diet on the expression of GPR120 revealed that after 3 weeks and 6 months the level of mRNA for GPR120 in the tissue was significantly increased which coincided with and probably reflected a significant increase in the number of GPR120 positive cells in the corpus region; in contrast, within the antrum region, the number of GPR120 cells decreased. Furthermore, dietary fat intake also led to changes in the number of enteroendocrine cells producing either ghrelin or gastrin. After 3 weeks and even more pronounced after 6 months the number of ghrelin cells and gastrin cells was significantly increased. These results imply that a HF diet leads to significant changes in the cellular repertoire of the stomach mucosa. Whether these changes are a consequence of the direct exposure to HF in the luminal content or a physiological response to the high level of fat in the body remains elusive.

## Introduction

The gastrointestinal system is supposed to play an important role in the development of diet induced obesity (DIO) (Bray, 2004; Hyland et al., 2010) which has been attributed to a disruption or alteration of intestinal signaling (Duca et al., 2013a). So far, the attention of research in this field was mainly concentrated on hormones from the small intestine, such as plasma cholecystokinin (CCK), glucagon-like peptide-1 (GLP-1), or peptide YY (PYY) and alterations which coincide with high fat (HF) feeding including changes in gastrointestinal (GI) hormone secretion and impacts on the regulation of GI motility responses (Covasa and Ritter, 2000; le Roux et al., 2006; Williams et al., 2011). Very little is known about changes in the stomach due to DIO and there remains a paucity of data about diet induced alterations induced by HF diet, especially in the functionally distinct gastric compartments corpus and antrum. Recent studies analyzing tissue samples from the gastric mucosa of morbidly obese patients have shown that the density of ghrelin cells was significantly increased (Maksud et al., 2011; Widmayer et al., 2012). Moreover, the expression level of a G protein coupled receptor responding to medium and long chain fatty acids, GPR120 (FFAR4) (Hirasawa et al., 2005; Tanaka et al., 2008; Matsumura et al., 2009; Cartoni et al., 2010), was clearly elevated (Widmayer et al., 2012). Whether these changes are cause or consequence of overweight is elusive. Feeding mice with a HF diet for an appropriated time period is considered a potent model for studying effects which coincide with the development of DIO. Therefore, in the present study, mice were fed with a diet which differs in fat content [chow fed (CF) mice with 9% calories from fat vs. high fat diet fed (HF) mice with 60% calories from fat]. After a feeding period of 3 weeks and 6 months, respectively, tissue samples from different regions of the stomach were assessed for nutrient sensing receptors, specifically the lipid receptors GPR120 and GPR43, and density of distinct cell types.

## Materials and methods

### Animals and nutritional experiments

Analyses were performed with wild type mouse strains C57/BL6J purchased from Charles River (Sulzfeld, Germany). After a weaning period for 4 weeks, four groups, each consisting of five male littermates, were nourished on diets with differing fat calorie contents for different feeding durations. Two control groups were fed a standard laboratory chow containing 9% calories from fat (CF) (3.06 kcal/g, 58% from carbohydrates and 33% from protein; V1534-300 R/M-H, ssniff Spezialitäten GmbH, Soest, Germany) and two HF groups a HF diet containing 60% calories from fat (5.24 kcal/g, 20% from carbohydrates and 20% from protein, D12492 Research diets), maintaining one of each group either for 3 weeks or 6 months on the respective diet. All mice were allowed free access to food and water. Body weight was determined weekly (final body weight: after 3 weeks: CF: 22.14 g ± 1.52, HF: 20.92 g ± 0.89; after 6 months: CF: 30.28 g ± 1.43, HF: 51.46 g ± 2.62). Before dissection of the stomach, mice were fasted for 5 h until 1 p.m. After the fasting period mice were killed via inhalation of lethal doses of carbon dioxide delivered by a compressed gas cylinder. All experiments comply with the Principles of Animal Care, publication no. 85-23, revised 1985, of the National Institutes of Health and with the current laws of Germany.

### Tissue preparation

After removal of the storage compartment fundus, the stomach was opened along the greater curvature and subdivided along the lesser curvature into two equal halves, each further separated into corpus and antrum in 1 × phosphate-buffered saline (PBS) (0.85% NaCl, 1.4 mM KH_2_PO_4_, 8 mM Na_2_HPO_4_, pH 7.4). For RNA isolation material was immediately transferred into a collection tube, frozen in liquid nitrogen and stored at −70°C until use and for immunohistochemical analyses immersion-fixed in 4% buffered paraformaldehyde in 150 mM phosphate buffer, pH 7.4) for either 15 min (for GPR120, TRPM5, ghrelin and gastrin) or 4 h (for GPR43) at 4°C followed by cryoprotection in 25% sucrose at 4°C overnight. Subsequently, gastric tissue was embedded in Leica OCT cryocompound tissue freezing medium (Leica Microsystems, Bensheim, Germany) and quickly frozen on liquid nitrogen. Longitudinal sections (8 μm) were cut on a CM3000 cryostat (Leica Microsystems, Bensheim, Germany) and attached to Superfrost Plus microslides (Menzel Gläser, Braunschweig, Germany).

### RNA isolation, cDNA synthesis, qPCR, cloning, and sequencing

Total RNA of frozen samples from CF and HF mice was prepared by the NucleoSpin RNA kit (Macherey-Nagel, Düren, Germany) according to the manufacturer's protocol. To ensure the complete removal of DNA, a DNase digestion (DNase I, Life Technologies, Carlsbad, CA, USA) step was included. Subsequently, 1.5 μg total RNA was reverse transcribed using oligo(dT) primers and SuperScript III Reverse Transcriptase (Invitrogen, Carlsbad, CA, USA). RNA integrity of each sample was confirmed by the amplification of the housekeeping gene for the ribosomal protein L8 with intron spanning primers to verify the DNA removal.

Real time PCR experiments were performed as previously described (Widmayer et al., 2012). In brief, changes in mRNA levels were performed using the Light Cycler (Roche Diagnostics, Mannheim, Germany). The qPCR reaction mixture (10 μl) consisted of 2 × KAPA SYBR Fast qPCR Master Mix (Peqlab Biotechnologie, Erlangen, Germany) and primer sets. Relative amounts of transcripts for GPR120 and GPR43 were normalized to the expression of L8 which remained the same in samples from HF and control mice. The following qPCR protocol was used: 95°C for 2 min, 95°C for 15 s, 60°C for 15 s, 72°C for 15 s with 40 cycles, a melting step by slow heating from 65 to 95°C with +0.5°C per cycle and a final cooling down to 40°C. Each assay included (in triplicate): for target genes 112.5 ng of each tested cDNA, for L8 a 1:10 cDNA dilution and a non-template control reaction. For efficiency acquirement, standard curves of serial dilutions (in steps of tenfold) of a calibrator cDNA ranging from 375 to 0.0375 ng were generated. LightCycler Software 3.5 (Roche Diagnostics) results were exported as tab-delimited text files and imported into Microsoft Excel for calculations of the expression ratios using the mean crossing points of target and reference genes from controls and samples. Efficiencies were acquired by the generation of standard curves of dilution series of a calibrator cDNA with each primer set and the given slopes in the LightCycler Software 3.5 (Roche Diagnostics). For the amplification of FA receptors and L8 the following intron spanning primers were used: GPR120 primers, (nt 700–822 from GenBank accession number NM_181748; the expected size of PCR products, 123 bp) 5′-GTG CCG GGA CTG GTC ATT GTG-3′ and 5′-TTG TTG GGA CAC TCG GAT CTG G-3′; GPR43 primers, 5′-AAC TCG GGA TGC TTC AGC CTG-3′ and 5′-AGA TGG GGG GAA AGG TGT AGG G-3′ (NM_146187, nt 255–529, 275 bp), and L8 primers, 5′-GTG CCT ACC ACA AGT ACA AGG C-3′ and 5′-CAG TTT TGG TTC CAC GCA GCC G-3′ (BC043017, nt 548–771, 224 bp, 375 bp genomic contamination).

The amplified PCR products were cloned into pGEM-T vector (Promega, Medison, WI, USA) and validated by DNA sequencing using an ABI Prism 310 Genetic Analyzer (Applied Biosystems, Foster City, CA, USA). Partial sequences were matched to corresponding mouse full-length sequences by GenBank BLAST searches.

### Immunohistochemistry

Cryosections were air-dried, rinsed in 1 × PBS for 10 min and incubated with blocking solution [1 × PBS with 10% normal donkey serum (NDS), 0.3% Triton X-100] for 30 min at room temperature. Then, the blocking solution was replaced by the primary antibody diluted in 1 × PBS containing 10% NDS and 0.3% Triton X-100 at 4°C overnight. Antibodies were used in the following dilutions: rabbit anti-GPR120 (SAB4501490, Sigma Aldrich, Steinheim, Germany) 1: 200, rabbit anti-TRPM5 (Kaske et al., 2007) 1:800, goat anti-ghrelin (sc-10368; Santa Cruz Biotechnology, Santa Cruz, CA, USA) 1:2000, guinea pig anti-gastrin (BP5046, Acris Antibodies, Herford, Germany) 1:2000, mouse anti-acetylated-α-tubulin antibody (Sigma Aldrich) 1:100, rabbit anti-chromogranin A (CgA) antibody (no. 20086, Immunostar, Hudson, WI, USA) 1:50, goat anti-GPR43 1:200 (sc-28424; Santa Cruz). Specificity and use of the antibodies were documented elsewhere (TRPM5: Kaske et al., 2007; ghrelin: Caminos et al., 2003; gastrin: Fischer et al., 2008; acTub: Saqui-Salces et al., 2011; CgA: Cui et al., 2004, GPR43: Eberle et al., 2014). Due to a recent report by Janssen et al. (2012), specificity of the GPR120 antibody was assayed in the gastric groove and duodenum where brush cells at the limiting ridge as well as some ghrelin cells in the small intestine were positively stained. In addition, control experiments on consecutive tissue sections were performed in which the respective primary antibody was omitted. After washing in 1 × PBS, bound primary antibodies were visualized using appropriate secondary antibodies conjugated to Alexa 488, Alexa 568, or Cy3 (Invitrogen, Karlsruhe, Germany; 1:500 in blocking solution) for 2 h at room temperature. After three rinses for 5 min in 1 × PBS, sections were counterstained with 4′, 6-diamidino-2-phenylindole (DAPI)-containing solution (1 μg/ml in 1 × PBS, Sigma Aldrich, Schnelldorf, Germany) for 3 min at room temperature to visualize nuclei, then rinsed in bidest and finally mounted in Mowiol (Roth, Karlsruhe, Germany). No immunoreactivity could be observed when the primary antibodies were omitted.

### Microscopy and imaging

Immunofluorescence was examined and documented with a Zeiss Axiophot microscope (Carl Zeiss MicroImaging, Jena, Germany). Images were captured using a SensiCam CCD camera (PCO Computer Optics, Kelheim, Germany), adjusted for contrast in AxioVision LE Rel. 4.3 (Carl Zeiss MicroImaging, Jena, Germany) and arranged in PowerPoint (Microsoft) or Adobe Photoshop (Adobe Systems, San Jose, CA, USA).

### Cell quantification

Cell counting was conducted as previously described (Widmayer et al., 2012). Briefly, random sampling fields of longitudinally cutted sections through the proximal corpus and antral compartment were selected above the lamina muscularis (GPR43, ghrelin, gastrin) or in the upper mucosal half (GPR120, TRPM5) where the respective immunopositive cells resided. To determine the number of cells, microscopic digital images of four to five consecutive sections were acquired by defining sampling fields of 320 × 260 μm. Cell counting was performed in a blind manner, and the average cell counts of immunopositive cells per field were determined. Immunoreactive cells were only counted when the nuclei were clearly visible by DAPI staining.

### Statistical analysis

For the quantification of relative changes in mRNA expression levels, data (in triplicate) were expressed as mean fold differences ± S.D. compared with that of controls, with values = 1 representing the baseline level corresponding to no relative difference in expression levels. The formula used to calculate the n-fold difference of mRNA expression levels of target genes relative to that of reference genes was as follows: ratio = (E_target)^ΔCt target (control−sample)^ : (E_ref_)^ΔCt ref (control−sample)^_. For determination of cell numbers, values were given as mean ± S.D. for the quantification of four to five consecutive sections. Significant differences between the groups were analyzed by the unpaired *t*-test with GraphPad Prism (Graphpad Software, www.graphpad.com). Statistical significance was set at *P* < 0.05.

## Results

### Effect of HF feeding on body weight

**Figure 1:**
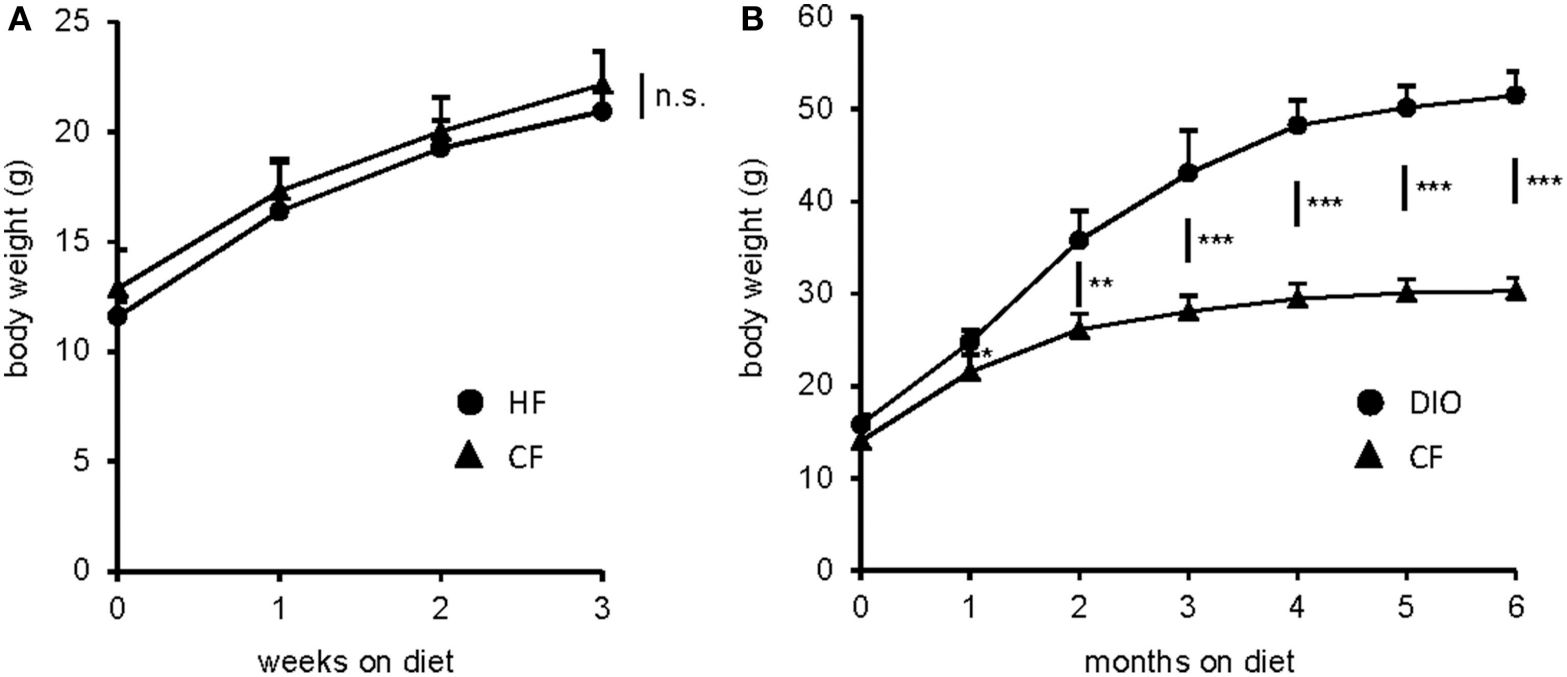
**Body weights after standard and HF diet feeding for different time periods**. **(A)** After 3 weeks fed with the HF diet, body weight of CF, and HF mice was almost comparable (*n* = 5, each). **(B)** HF diet feeding for 6 months resulted in excess body weight of DIO mice relative to CF controls (*n* = 5, each). *P*-values were calculated using the unpaired *t*-test. Data presented as mean ± S.D. Statistically significant results are indicated by ^*^P < 0.05, ^**^P < 0.005, ^***^P < 0.0001 and n.s., not significant.

To monitor possible changes induced by long-term consumption of a diet enriched in long chain fatty acids, animals were subjected to a standard diet (CF) and to a HF diet (60% kcal from fat), respectively. To generate DIO models, diets were introduced immediately after the weaning period of 4 weeks and fed for 3 weeks and 6 months, respectively. During the first 3 weeks, it was observed that CF and HF mice almost equally gained weight and did not differ in body weight (Figure 1A). Similar results were observed in DIO models (Chen et al., 2010; Duca et al., 2013b), although other studies reported an impact on body weight already after 1 week of HF diet (Winzell and Ahrén, 2004; Nefti et al., 2009). The discrepancy may arise from gender, age or animal models used. After 4 weeks, weight gain was progressively higher in HF diet fed mice and reached a plateau in the course of HF feeding (Figure 1B). When exposed to the HF diet for 6 months, a marked gain in body weight was estimated. HF mice increased their weight by 70% than control mice.

### Effect of HF feeding on the expression of fatty acid receptors and the number of GPR120 and ghrelin cells in the corpus

**Figure 2:**
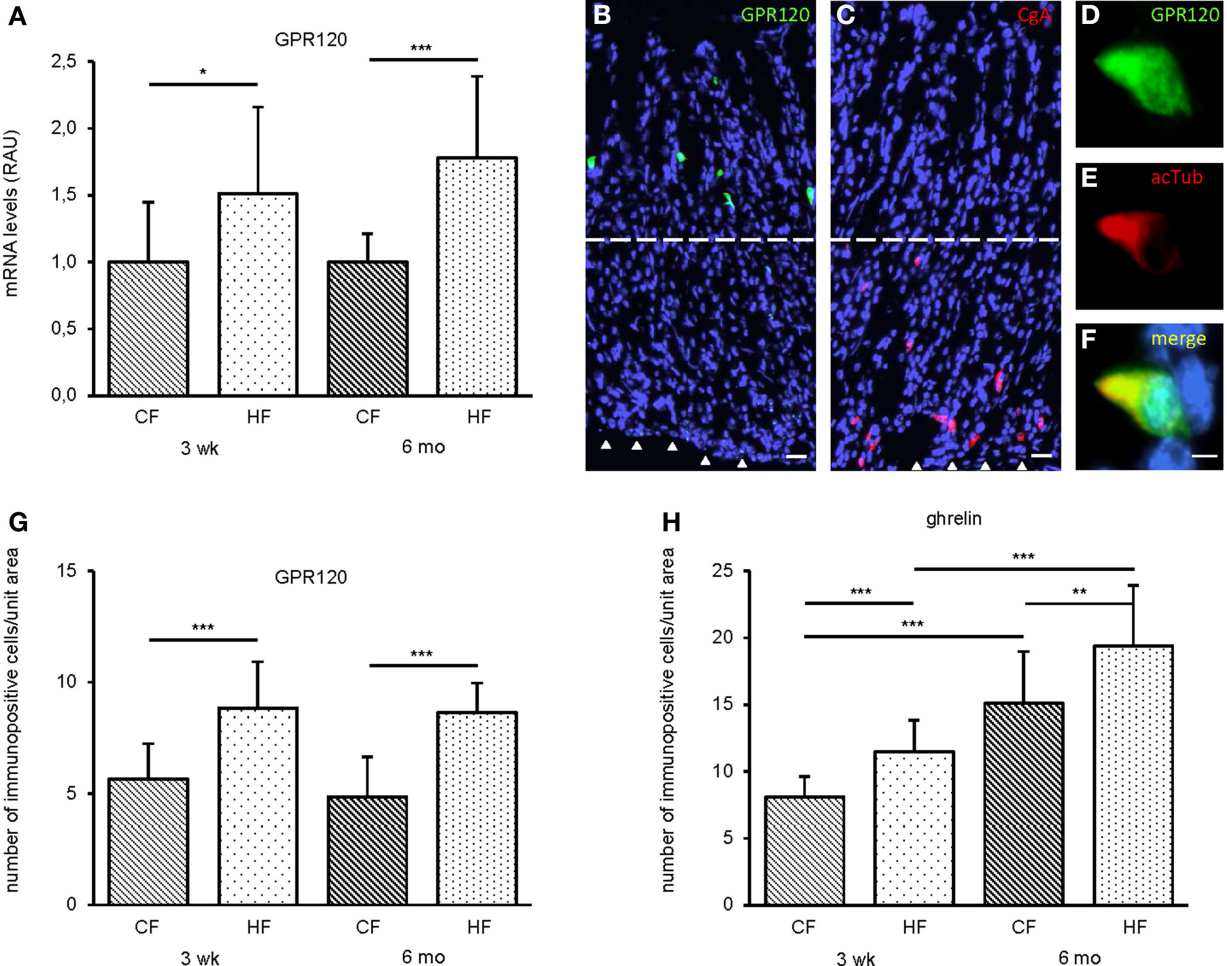
**Impact of HF feeding and feeding duration on relative mRNA amounts for the LCFA receptor GPR120 and numbers of GPR120 and ghrelin cells in the corpus of CF and HF mice**. **(A)** Relative expression levels for GPR120 were measured using real time PCR (*n* = 5, each group). Feeding a HF diet for 3 weeks already increased GPR120 mRNA levels 1.5 fold in HF mice (dotted) relative to controls (hatched). After 6 months mRNA amounts increased 1.8 times. Data are expressed in relative arbitrary units (RAU) as mean ± S.D and generated in triplicate. Statistically significant results determined by the unpaired *t*-test are indicated by ^*^P < 0.05, ^**^P < 0.005, ^***^P < 0.0001, and n.s., not significant. **(B)** In longitudinal sections through the oxyntic mucosa GPR120 immunoreactive cells were restricted to the upper half. **(C)** CgA positive cells were located in the lower portion of the gastric epithelium. Hatched lines separate the gastric apical and basal halves and arrowheads mark the basement membrane of the gastric mucosa. Sections were counterstained with DAPI. Scale bar **(B,C)**, 20 μm. **(D–F)** Double immunolabeling analyses demonstrate the colocalization of GPR120 **(D)** and the brush cell marker acTub **(E)**, as shown in the merged image **(F)** scale bar **(D–F)**, 5 μm **(G)** Quantification of GPR120 positive cells upon a HF diet for 3 weeks and 6 months. In the apical half of the corpus mucosa immunoreactive cells for GPR120 were counted in a unit area of 320 × 260 μm on four to five consecutive longitudinal sections. Mean numbers (± S.D.) of immunopositive cells in CF (hatched) and HF (dotted) mice (*n* = 5, each). Cell densities are expressed as labeled cell numbers per unit area. **(H)** Both short- and long-term feeding a HF diet increased the frequency of ghrelin cells in the corpus.

To approach the question whether feeding a HF diet might have an impact on the expression of sensors for long chain fatty acids in the gastric mucosa, as a first step, the amount of mRNA for GPR120, a receptor for LCFAs (Hirasawa et al., 2005; Tanaka et al., 2008), was determined by real time PCR experiments. Tissue samples from the corpus mucosa of HF mice and from control mice were analyzed. In mice fed the HF diet for 3 weeks, the level of mRNA for GPR120 was significantly increased (*P* = 0.0059), and similarly in mice fed for 6 months (*P* < 0.0001) (Figure 2A). For comparison, the level of mRNA for the receptor GPR43 (FFAR2) which responds to short chain fatty acids was assessed. It was found that in mice fed with HF diet for 3 weeks the mRNA level for GPR43 was not changed (0.88 ± 0.50, *P* = 0.3202).

The increase of mRNA levels for the receptor GPR120 during HF diet for a few weeks or months could either reflect more transcripts per cell or alternatively could be due to a higher number of cells expressing the LCFA receptor GPR120. To determine the number and identity of cells expressing GPR120, tissue sections through samples from the proximal corpus were analyzed using a specific antibody for GPR120. The immunohistochemical procedure resulted in strongly labeled cells predominately scattered throughout the upper portions of oxyntic glands, where enteroendocrine (EE) cells rarely reside (Ku et al., 2003). The morphology and scattered distribution of these cells in the mucosa are reminiscent of brush cells located in the pit region of gastric glands which belong to a defined lineage of epithelial cells (Saqui-Salces et al., 2011, 2012). In longitudinal sections of the corpus this cell type was easily identifiable due to its GPR120 labeling and segregation to upper portions of gastric glands (Figure 2B). In contrast, EE cells are positioned at the base of gastric glands; Figure 2C shows the distribution pattern of these cells using the chromogranin A antibody specific for EE cells. Based on the notion that labeling with an acetylated-α-tubulin (acTub) antibody is indicative for brush cells (Saqui-Salces et al., 2011), double immunohistochemical analyses confirmed a colocalization with GPR120 (Figures 2D–F). Furthermore, staining experiments with the GPR120 antibody resulted in weakly labeled cells in the lower half of the oxyntic mucosa (data not shown); these cells represent probably ghrelin cells which seem to express GPR120 (Engelstoft et al., 2013). For a quantification of the immunoreactive cells in defined areas, random sampling fields of 320 × 260 μm were selected and stained cells located in the upper half of the mucosa determined; the few very faintly labeled cells located near the epithelial base were not included. The data collected for tissue samples from control mice and for HF mice revealed clear differences between both groups (CF: 5.7 ± 1.6, HF: 8.9 ± 2.1, *P* < 0.0001) (Figure 2G). The number of GPR120 expressing cells in a defined area was about 1.6 times higher in mice fed with HF for 3 weeks than in control mice. After 6 months on HF feeding, approximately the same numbers of GPR120 cells were counted (CF: 4.9 ± 1.8, HF: 8.7 ± 1.3), representing a 1.8 fold increase (*P* < 0.0001). The increase in the number of GPR120 cells coincided with the rise in the number of TRPM5 cells. Within the upper half of the oxyntic glands after 3 weeks the number of TRPM5 cells was increased 1.6 fold (CF: 6.2 ± 1.8, HF: 9.8 ± 2.0, *P* < 0.0001) and after 6 months 1.8 fold (CF: 5.2 ± 1.2, HF: 9.3 ± 2.8, *P* < 0.0001). Staining for GPR43 led to strongly labeled cells predominately located in the basal portions of gastric glands which in double immunohistochemical analyses with chromogranin A were identified as enteroendocrine cells (data not shown). Upon a feeding period of 3 weeks, the number of basally located cells expressing the SCFA receptor GPR43 remained unaffected (CF: 8.5 ± 2.1, HF: 8.0 ± 2.0, *P* = 0.4908).

It has previously been reported that dietary fat, in particular linolenic acid, has a strong impact on the secretion of ghrelin (Janssen et al., 2012; Lu et al., 2012; Gong et al., 2014), therefore we next examined whether the number of ghrelin cells in the corpus region may change upon feeding of a HF diet. The results indicate that the population of ghrelin cells expanded during the periods of HF feeding. Already after 3 weeks, the number of ghrelin cells was increased (CF: 8.1 ± 1.5, HF: 11.5 ± 2.4, 1.4 fold increase, *P* < 0.0001) (Figure 2H). After 6 months, the number of ghrelin cells was increased to 15.1 ± 3.9 cells in control mice and 19.4 ± 4.5 in HF mice (1.3 fold increase, *P* = 0.0026) (Figure 2H).

### Effect of HF feeding on the expression of fatty acid receptors and the number of GPR120 and gastrin cells in the antrum

**Figure 3:**
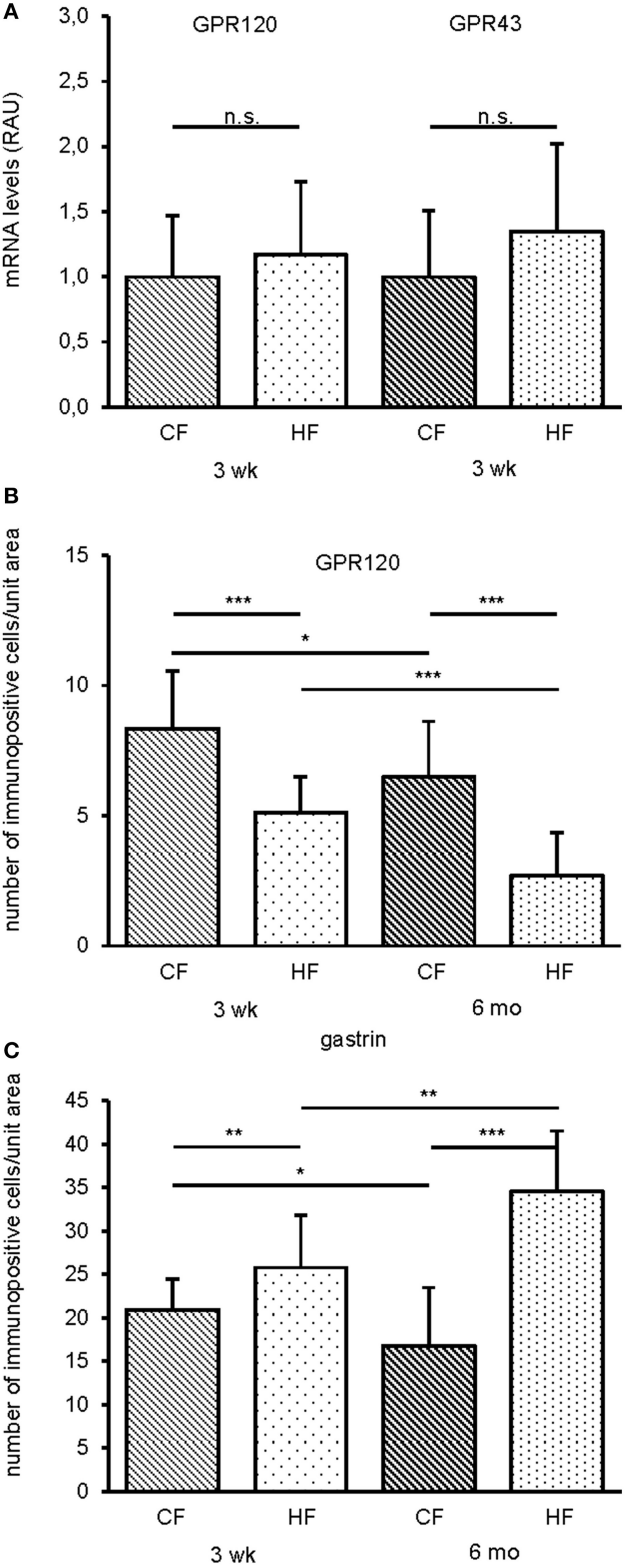
**Effect of HF feeding on relative mRNA amounts of fatty acid receptors and numbers of GPR120 and gastrin cells in the antrum of CF and HF mice**. **(A)** Feeding a HF diet for 3 weeks did not change mRNA levels for GPR120 and GPR43. HF mice (dotted), controls (hatched). Real time PCR data are expressed in relative arbitrary units (RAU) as mean ± S.D and generated in triplicate (*n* = 5, each). **(B)** Frequency of GPR120 immunoreactive cells decreased in the antrum of HF fed mice after 3 weeks and 6 months, respectively. **(C)** After 3 weeks and 6 months density of gastrin cells is elevated upon a HF diet in the antrum. Mean numbers (± S.D.) of immunopositive cells in CF (hatched) and HF (dotted) mice (*n* = 5, each). Cell densities are expressed as labeled cell numbers per unit area. ^*^P < 0.05, ^**^P < 0.005, and ^***^P < 0.0001, n.s., not significant.

For the distal glandular compartment of the stomach, the antrum, it was found that after a feeding period of 3 weeks both mRNA levels for GPR120 and GPR43 were unchanged (Figure 3A). However, there was a significant change in the number of GPR120 immunopositive cells in the apical half of the antrum. After the feeding period of 3 weeks, it was found that the number of GPR120 was significantly decreased (CF: 8.4 ± 2.2, HF: 5.1 ± 1.4, 0.6 fold decrease, *P* < 0.0001) (Figure 3B). Likewise, quantification of TRPM5 cells revealed similar results (CF: 9.1 ± 3.5, HF: 6.4 ± 2.0, 0.7 fold decreased, *P* = 0.00041). After 6 months the number of labeled cells was even lower (GPR120: CF: 6.5 ± 2.1, HF: 2.7 ± 1.7, 0.4 fold decrease, *P* < 0.0001; TRPM5: CF: 6.1 ± 2.3, HF: 3.2 ± 1.2, 0.5 fold decrease, *P* < 0.0001) (Figure 3B). In contrast, the number of GPR43 cells in the basal half remained unchanged (CF: 1.9 ± 1.4, HF: 1.4 ± 1.2, *P* = 0.1825). These results indicate that in the antrum compartment the number of apically located GPR120 and TRPM5 cells significantly decreased in the course of HF feeding.

Since lipids, specifically medium chain triacylglycerol, seem to enhance the secretion of gastrin (Furuse and Dockray, 1995), the number of gastrin cells in the antrum was quantified. After a HF diet for 3 weeks, the number of gastrin cells was increased 1.2 fold (CF: 21.0 ± 3.5, HF: 25.8 ± 6.0, *P* = 0.0001) (Figure 3C). After 6 months, the difference between control and HF mice was significantly more pronounced than after 3 weeks (CF: 16.8 ± 6.7, HF: 34.6 ± 7.0, 2 fold increase, *P* < 0.0001) (Figure 3C).

Taken together the present results indicate that particular cell types in the gastric mucosa respond to HF feeding and high supply of LCFAs with distinct changes in the density of enteroendocrine and brush cells.

## Discussion

A balanced supply of dietary fat to the alimentary tract is associated with well adapted physiological processes including the appropriate regulation of peptide hormone secretion, while excessive intake of fats leads to disordered responses. In the current study we have assessed how a HF diet enriched in LCFAs may affect fatty acid receptors in the gastric mucosa of non-obese and obese mice. The results indicate that in the corpus feeding a HF diet led to elevated amounts of mRNA for the receptor GPR120 which is responsive to long chain fatty acids (Hirasawa et al., 2005; Tanaka et al., 2008). This effect was apparently independent of body weight gain. For the receptor GPR43 which is responsive to short chain fatty acids (Brown et al., 2003) the mRNA levels in the corpus remained unchanged. These results imply that the expression of GPR120 is particularly sensitive to an excess supply of long chain fatty acids in the diet. An enhanced expression of GPR120 was recently also observed in the small intestine, when DIO rats were fed a HF diet (Duca et al., 2013b). An upregulation of GPR120 expression could improve the responsiveness of cells to LCFAs, however, alterations in mRNA levels might also reflect changes in the number of cells expressing GPR120. In fact, in the corpus we have found that the dominating population of GPR120 positive cells was increased per unit area. The segregation of the cells to the upper mucosal half, as well as the cellular shape and the presence of acetylated-α-tubulin define GPR120 expressing cells as brush cells (Saqui-Salces et al., 2011, 2012) whose function thus be directly influenced by the ingested lipids in the gastric lumen. A possible role in fatty acid sensing by GPR120 might be important for the local regulation of fat digestive processes, e.g., for an appropriate production of gastric lipase secreted by gastric chief cells (Liao et al., 1983). It is conceivable that the presence of lipids affects the gastric lipase levels by an activation of chief cells via signals transmitted from the sensing of brush cells, possibly via paracrine cell-cell communication (Eberle et al., 2013).

A higher number of cells was observed after 3 weeks and persisted for 6 months. The reason why more GPR120 cells per unit area exist remains unclear. These changes could either result from the generation of additional cells as a consequence of proliferation and differentiation from gastric epithelial progenitor cells (Modlin et al., 2003) or could be due to a reduced apoptosis rate resulting in a longer life span of mature cells. In fact, LCFAs seem to enhance cell survival, as has been shown for the murine enteroendocrine cell line STC-1 and this effect appeared to be mediated by GPR120 (Katsuma et al., 2005). Both proliferative and apoptotic pathways are known to be susceptible to various influences. Since the gastric mucosa is in constant renewal, modifications in the amount and content of food might represent a suitable tool to manipulate mucosal cell composition (Larsson, 2000).

HF feeding caused different effects in the two stomach compartments corpus and antrum; there was a rise in the number of GPR120 cells in the corpus and a decrease in the number of GPR120 cells in the antrum. The reason for the opposed effect and its consequences remain elusive, however, apparently reflect adaptive responses due to compartment-specific requirements. In fact, it has been shown that gastric emptying was accelerated in DIO animals (Covasa and Ritter, 2000), while the antrum motility was attenuated (Boyd et al., 2003).

By determining the number of ghrelin and gastrin cells we have shown that also these EE cell populations were expanded during the course of HF feeding. The increase in the number of ghrelin cells is in line with previous reports indicating correlation of obesity and ghrelin cell density in human (Maksud et al., 2011; Widmayer et al., 2012). This observation is remarkable since reduced levels in circulating ghrelin are found in obese individuals (Tschöp et al., 2001; Beck et al., 2002; Lee et al., 2002). In this context, it is interesting to note that LCFAs inhibit the release of ghrelin, an effect that appears to be mediated by the activation of GPR120 (Janssen et al., 2012; Lu et al., 2012; Gong et al., 2014). Thus, it is conceivable that the increased number of ghrelin cells might reflect a compensatory response to a reduced secretory capacity of ghrelin cells following a sustained GPR120-mediated inhibitory effect of fatty acids on ghrelin secretion. The enlarged number of gastrin cells found in mice fed on HF diet may account for the higher circulating gastrin levels that have been measured in HF diet fed mice (Saqui-Salces et al., 2012). This notion is in line with the findings that gastrin secretion is regulated by medium chain triacylglycerol as potent stimulators (Furuse and Dockray, 1995) and that gastrin cells are equipped with lipid sensors (hr, Pedersen, Gille, Egerod, Engelstoft and Husted, Nøhr et al., 2013; Widmayer, unpublished observation).

In conclusion, in the compartment corpus feeding mice with a HF diet resulted in increased mRNA levels of the receptor GPR120 which is responsive to long chain fatty acids. These changes are associated with an increased number of GPR120 cells. Changes in the number of cells were also found for EE cells which produce the peptide hormones ghrelin and gastrin. The changes in cell number might be a reaction of adequately adjusting gastric secretory activities and signaling processes to the lipid content in the interstitial lumen and/or the body fluid. Such alterations may precede the onset of obesity and/or accompany its progression.

### Conflict of interest statement

The authors declare that the research was conducted in the absence of any commercial or financial relationships that could be construed as a potential conflict of interest.

## Abbreviations

CF: Chow fed
DAPI: 4,6-diamidino-2-phenylindole
DIO: Diet induced obesity
FFAR: Free fatty acid receptor
GI: Gastrointestinal
GLP-1: Glucagon-like peptide-1
HF: High fat
LCFA: Long chain fatty acid
PYY: Peptide tyrosine tyrosine
qPCR: Quantitative polymerase chain reaction
TRPM5: Transient receptor potential cation channel, subfamily M, member 5.
